# Evaluating the oral delivery of GalNAc-conjugated siRNAs in rodents and non-human primates

**DOI:** 10.1093/nar/gkae350

**Published:** 2024-05-14

**Authors:** Mikyung Yu, June Qin, Xiumin Liu, Diane Ramsden, Brian Williams, Ivan Zlatev, Dale Guenther, Shigeo Matsuda, Roxanne Tymon, Justin Darcy, Catrina Wong, Jamie Tsung, Peter Zawaneh, Saeho Chong, Christopher S Theile, Nathan Taneja, Arlin Rogers, Ju Liu, Elena Castellanos-Rizaldos, Sarah Bond, Kawai So, Jason Denoncourt, Adam Castoreno, Muthiah Manoharan, Jing-Tao Wu, Kevin Fitzgerald, Martin A Maier, Vasant Jadhav, Jayaprakash K Nair

**Affiliations:** Alnylam Pharmaceuticals, Inc., Cambridge, MA 02142, USA; Alnylam Pharmaceuticals, Inc., Cambridge, MA 02142, USA; Alnylam Pharmaceuticals, Inc., Cambridge, MA 02142, USA; Alnylam Pharmaceuticals, Inc., Cambridge, MA 02142, USA; Alnylam Pharmaceuticals, Inc., Cambridge, MA 02142, USA; Alnylam Pharmaceuticals, Inc., Cambridge, MA 02142, USA; Alnylam Pharmaceuticals, Inc., Cambridge, MA 02142, USA; Alnylam Pharmaceuticals, Inc., Cambridge, MA 02142, USA; Alnylam Pharmaceuticals, Inc., Cambridge, MA 02142, USA; Alnylam Pharmaceuticals, Inc., Cambridge, MA 02142, USA; Alnylam Pharmaceuticals, Inc., Cambridge, MA 02142, USA; Alnylam Pharmaceuticals, Inc., Cambridge, MA 02142, USA; Alnylam Pharmaceuticals, Inc., Cambridge, MA 02142, USA; Alnylam Pharmaceuticals, Inc., Cambridge, MA 02142, USA; Alnylam Pharmaceuticals, Inc., Cambridge, MA 02142, USA; Alnylam Pharmaceuticals, Inc., Cambridge, MA 02142, USA; Alnylam Pharmaceuticals, Inc., Cambridge, MA 02142, USA; Alnylam Pharmaceuticals, Inc., Cambridge, MA 02142, USA; Alnylam Pharmaceuticals, Inc., Cambridge, MA 02142, USA; Alnylam Pharmaceuticals, Inc., Cambridge, MA 02142, USA; Alnylam Pharmaceuticals, Inc., Cambridge, MA 02142, USA; Alnylam Pharmaceuticals, Inc., Cambridge, MA 02142, USA; Alnylam Pharmaceuticals, Inc., Cambridge, MA 02142, USA; Alnylam Pharmaceuticals, Inc., Cambridge, MA 02142, USA; Alnylam Pharmaceuticals, Inc., Cambridge, MA 02142, USA; Alnylam Pharmaceuticals, Inc., Cambridge, MA 02142, USA; Alnylam Pharmaceuticals, Inc., Cambridge, MA 02142, USA; Alnylam Pharmaceuticals, Inc., Cambridge, MA 02142, USA; Alnylam Pharmaceuticals, Inc., Cambridge, MA 02142, USA

## Abstract

Oral delivery is the most widely used and convenient route of administration of medicine. However, oral administration of hydrophilic macromolecules is commonly limited by low intestinal permeability and pre-systemic degradation in the gastrointestinal (GI) tract. Overcoming some of these challenges allowed emergence of oral dosage forms of peptide-based drugs in clinical settings. Antisense oligonucleotides (ASOs) have also been investigated for oral administration but despite the recent progress, the bioavailability remains low. Given the advancement with highly potent and durable trivalent *N*-acetylgalactosamine (GalNAc)-conjugated small interfering RNAs (siRNAs) *via* subcutaneous (s.c.) injection, we explored their activities after oral administration. We report robust RNA interference (RNAi) activity of orally administrated GalNAc–siRNAs co-formulated with permeation enhancers (PEs) in rodents and non-human primates (NHPs). The relative bioavailability calculated from NHP liver exposure was <2.0% despite minimal enzymatic degradation in the GI. To investigate the impact of oligonucleotide size on oral delivery, highly specific GalNAc-conjugated single-stranded oligonucleotides known as REVERSIRs with different lengths were employed and their activities for reversal of RNAi effect were monitored. Our data suggests that intestinal permeability is highly influenced by the size of oligonucleotides. Further improvements in the potency of siRNA and PE could make oral delivery of GalNAc–siRNAs as a practical solution.

## Graphical Abstract

**Graphical Abstract:**
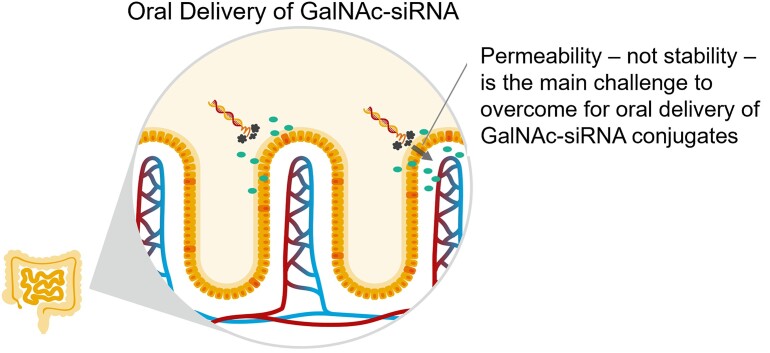

## Introduction

Oral administration of medicines offers patients painless and convenient mode of treatment and has been in practice for a long time. Unlike small molecule-based drugs, the oral delivery of hydrophilic agents greater than 1 kDa remains challenging because of poor intestinal permeability and susceptibility to degradation in the harsh environments of gastrointestinal (GI) tract with the extreme pH gradient and metabolic enzymes (1,2). Accordingly, approved biologics and nucleic acid-based therapeutics are typically administered through parental routes such as intravenous (i.v.), subcutaneous (s.c.) and intramuscular (i.m.) injections.

Nucleic acid-based therapeutics including antisense oligonucleotides (ASOs), small interfering RNAs (siRNAs) and modified messenger RNAs (mRNAs) are rapidly growing class of medications for the treatment and prevention of a wide range of diseases (3). siRNAs have demonstrated their ability to effectively and safely down-regulate disease-associated mRNAs by utilizing the endogenous RNA interference (RNAi) pathway (4) and represent a clinically validated new class of medicines for both rare and prevalent diseases with six approved drugs and dozens of programs in clinical development (5,6). Despite their large size, highly anionic and hydrophilic nature, several studies evaluating oral delivery of siRNA in rodents have been reported previously but with limited success. For example, orally administered tumor necrosis factor alpha (TNF-α)–siRNA formulated with reactive oxygen species-responsive thioketal polymeric nanoparticles showed therapeutic potential in mice with dextran sodium sulphate-induced ulcerative colitis by reducing mRNA levels of TNF-α in inflamed intestinal tissues (7). Recently, bovine milk-derived exosome was reported as a potential oral delivery platform for siRNA and exhibited efficient *in vitro* intestinal permeability (8). However, one of the challenges in these nanoparticle-mediated oral delivery strategies is to ensure that the nanoparticle system maintains physicochemical properties as designed for the cytosolic delivery of siRNA to target cells, as well as overcomes biological hurdles in the GI track to achieve successful delivery efficiency after oral administration.

Considerable progress has been made for oral delivery of hydrophilic macromolecules which are smaller in size than siRNAs in the last few years. Oral semaglutide received U.S. Food and Drug Administration (FDA) approval in 2019, becoming the first oral glucagon-like peptide-1 receptor agonist (GLP-1 RA, 4114 Da) to treat type 2 diabetes (9). Less than a year later, the oral formulation of the somatostatin analog octreotide (1019 Da) was approved in 2020 for the treatment of acromegaly (10). These recent successful oral dosage forms of macromolecules utilize permeation enhancers (PEs) such as medium-chain fatty acids, or pH-sensitive polymers for enteric coating to improve GI absorption. More importantly, high potency and prolonged half-life (*t*_1/2_) of the macromolecular drugs in these delivery platforms enabled significant advancement of oral dosage form. Using this approach, chemically modified single stranded ASOs (∼7000 Da), larger than the oral peptide drugs in clinic, were formulated with enteric-coated tablets and demonstrated feasibility of the oral delivery across species. However, despite the progress in preclinical and clinical evaluations, currently there is no active clinical trial for the oral administration of an oligonucleotide drug (11–13).

Advances in siRNA design, including the development of the trivalent *N*-acetylgalactosamine (GalNAc)-conjugated delivery platform and chemical modifications to confer metabolic stability against nuclease degradation, have led to significant improvements in potency and duration of action. Conjugation of GalNAc ligand to siRNA facilitates efficient delivery to hepatocytes by targeting asialoglycoprotein receptors (ASGPR), which are highly expressed on the surface of hepatocytes (14–16). Thus, current pipelines of the RNAi therapeutics targeting mRNAs expressed in liver are dominated by fully chemically modified GalNAc–siRNAs which display improved pharmacokinetic/pharmacodynamic (PK/PD) and safety profiles (4,17). These favorable characteristics such as enhanced potency, metabolic stability, and prolonged duration of action provide an opportunity to revisit the oral delivery of siRNAs *via* approaches such as coformulation with PEs.

Here, we evaluated the oral administration for GalNAc–siRNAs formulated with various PEs in rodents and non-human primates (NHPs). We explored several PEs to identify combinations which may be suitable for oral siRNA delivery. These PEs including sodium caprate (C10) enabled oral absorption of GalNAc–siRNAs but showed overall low bioavailability. Our findings indicate that enzymatic degradation of GalNAc–siRNAs in the GI is minimal due to the improved metabolic stability of the chemically fully modified siRNAs. To further understand the mechanisms governing bioavailability, we examined the impact of oligonucleotide size on GI permeability. Previously, we have reported a highly specific GalNAc-conjugated single-stranded oligonucleotide, named REVERSIR, for rapid reversal of siRNA activity to enable control of RNAi pharmacology (18). We employed REVERSIRs with differing lengths to evaluate impact of oligonucleotide size on absorption in the GI when orally administered. We find that longer-length REVERSIR molecules require a higher oral dose than shorter versions to show comparable activity to the same REVERSIR administered *via* s.c. injection, suggesting lower bioavailability for the larger-sized molecules. This highlights that low intestinal permeability, which is attributed to the size and rigidity of double stranded siRNA molecules, rather than degradation in the GI remains to be the key limiting factor for their efficient oral delivery.

## Materials and methods

### Synthesis of GalNAc–siRNAs and REVERSIR oligonucleotides

GalNAc–siRNAs were synthesized on MerMade 192 or MerMade 12 synthesizer according to previously published protocols (16). GalNAc-CPG (controlled pore glass) support was prepared and used as previously described. 5′-*O*-(4,4′-dimethoxytrityl)-2′-deoxy-2′-fluoro- and 5′-*O*-(4,4′-dimethoxytrityl)-2′-*O*-methyl-3′-*O*-(2-cyanoethyl-*N*,*N*-diisopropyl) phosphoramidite monomers of uridine, 4-*N*-acetylcytidine, 6-*N*-benzoyladenosine and 2-*N*-isobutyrylguanosine were purchased commercially, and (*S*)-GNA phosphoramidites were synthesized according to previously published protocols (16). The phosphoramidite solutions were 0.15 M in anhydrous acetonitrile with 15% dimethylformamide (DMF) as a co-solvent for all monomers and coupled using standard conditions on the synthesizer (19). A solution of 0.6 M 5-(*S*-ethylthio)-1*H*-tetrazole in acetonitrile was used as the activator. Phosphorothioate linkages were introduced by sulfurization of phosphite linkages using 0.1 M 3-((*N*,*N*-dimethylaminomethylidene)amino)-3*H*-1,2,4-dithiazole-5-thione (DDTT) in pyridine. After completion of the solid-phase synthesis, the solid support was incubated in a sealed container with aqueous ammonium hydroxide (28–30%) and 5% diethylamine by volume. The mixture was kept in an incubator shaker overnight at 35°C (20). The solution was filtered to remove the support and washed with 5× volume of water. Liquid chromatography–mass spectrometry (LC–MS) and ion exchange (IEX) high-performance liquid chromatography (HPLC) were used to check deprotection and crude quality. IEX HPLC purification was then performed using mobile phase A (20 mM sodium phosphate in 15% acetonitrile) and mobile phase B (20 mM sodium phosphate + 1 M sodium bromide in 15% acetonitrile). TSKgel Super Q-5PW IEX resin (Tosoh, 0018546) was used for purification (21), and a general purification gradient of 15–48% in about 20 column volumes was applied. Fractions were analyzed by IEX HPLC using a Dionex DNAPac PA200 IEX analytical column (Thermo Fisher Scientific, 063000) at room temperature. A gradient of 30–50% mobile phase B over 12 min was employed at 1 ml/min. Fractions with >85% purity were pooled, concentrated, and desalted over size exclusion columns (GE Healthcare, 17-5087- 01) with a flow rate of 10 ml/min. The identities and purities of all oligonucleotides were confirmed using LC/electrospray ionization (ESI)-MS and IEX HPLC, respectively. The desalted siRNA single strands were further annealed to form siRNA duplexes as previously described (16).

**Figure 1.**
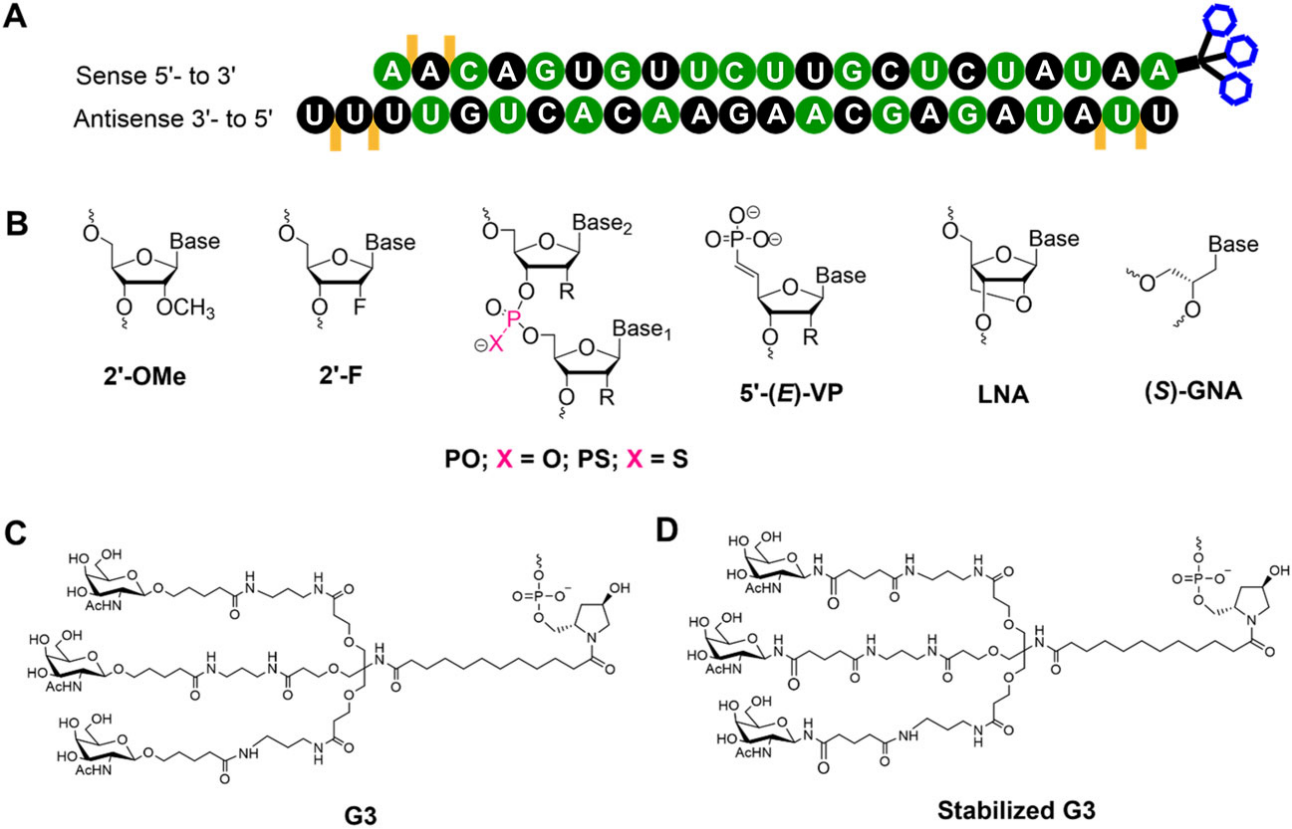
Schematic representation of siRNA and chemical modifications used in this study. (**A**) siRNA duplex. Color code denotes chemical modifications at 2′-position of the ribose moiety, linkage, and targeting ligand used in siRNA. Black circle, 2′-*O*-methyl modification; Green circle, 2′-deoxy-2′-fluoro modification; Yellow line, phosphorothioate linkage; Blue hexagon, *N*-acetylgalactosamine. (**B**) Structures of chemical modifications used in siRNA and REVERSIR compounds. Abbreviations: 2′-OMe = 2′-*O*-methyl; 2′-F = 2′-deoxy-2′-fluoro; PO = phosphodiester; PS = phosphorothioate; 5′-(*E*)-VP = 5′-(*E*)-vinylphosphonate; LNA = locked nucleic acid; (*S*)-GNA = (*S*)-glycol nucleic acid; G3 = GalNAc ligand. (**C, D**) Structures of G3 and stabilized G3.

REVERSIR oligonucleotides were synthesized on MerMade-12 DNA/RNA synthesizer at scales of 50–200 μmol. 2′-Deoxy 3′-phosphoramidites from Thermo and locked nucleic acid (LNA) 3′-phosphoramidites from Hongene were all used as received. A solution of 0.6 M 5-(*S*-ethylthio)-1H-tetrazole in acetonitrile was used as the activator. Phosphoramidite solutions were 0.15 M in anhydrous acetonitrile with 15% DMF as a co-solvent for 2′-OMe uridine and cytidine. The oxidizing reagent was 0.02 M I_2_ in THF/pyridine/water. *N*,*N*-Dimethyl-*N*′-(3-thioxo-3*H*-1,2,4-dithiazol-5-yl)methanimidamide (DDTT), 0.09 M in pyridine, was used as the sulfurizing reagent. The detritylation reagent was 3% dichloroacetic acid (DCA) in dichloromethane (DCM). After completion of the solid-phase synthesis, the CPG solid support was washed with 5% (v/v) piperidine in anhydrous acetonitrile three times with 5-min holds after each flow. The support was then washed with anhydrous acetonitrile and dried with argon. The oligonucleotides were then incubated with 28–30% (w/v) NH_4_OH at 35°C for 20 h. Crude oligonucleotide was collected by filtration, and the support was rinsed with water prior to analysis. Oligonucleotide solutions of approximately 1 OD_260_ units/ml were used for analysis of the crudes, and 30–50 μl of solution were injected. LC/ESI-MS was performed on an Agilent 6130 single quadrupole LC–MS system using an XBridge C8 column (2.1 × 50 mm, 2.5 μm) at 60°C with mobile phase A (200 mM 1,1,1,3,3,3-hexafluoro-2-propanol and 16.3 mM triethylamine in water) and mobile phase B (100% methanol). A gradient of 0–40% mobile phase B over 10 min was employed at 0.70 ml/min. All REVERSIR oligonucleotides were purified and desalted using previously reported methods (16). Representative siRNA and chemical modifications used in the study are shown in Figure 1. All siRNAs and REVERSIRs were synthesized by Alnylam Pharmaceuticals.

### PD studies in rodents

All studies were conducted using protocols consistent with local, state and federal regulations, as applicable, and were approved by the Institutional Animal Care and Use Committee (IACUC) at Alnylam Pharmaceuticals. The test article siRNAs (Table 1) for per oral (p.o.) and s.c. administrations were reconstituted in 75–150 mM C10 solution and phosphate-buffered saline (PBS), respectively. Female C57BL/6 mice (Charles River Laboratories) of 6–8 weeks old were fasted for 5 h as per the IACUC guidance and received oral gavage of test article siRNA solutions at a dose volume of 10 μl/g. Plasma samples were collected by using EDTA collection tubes and analyzed for target protein levels using an ELISA kit (F12: Innovative Research, Cat. No. IMSFXIIKTT; TTR: ALPCO, Cat. No. 41-PALMS-E01) in accordance with the manufacturer's protocol. Data were normalized to the pre-dose target protein level of individual animal or average of the pre-dose target protein levels for each group. For a comparison study in ASGPR knockout (KO) mice, both of female and male C57BL/6 wild-type (WT) mice (22 – 25 weeks old, Jackson Laboratories) and ASGPR KO mice (24–27 weeks old) supplied by in-house breeding colony were chosen, randomly assigned to each group, and given a single oral gavage of GalNAc–siRNA (siRNA-1) with C10. Fasting was introduced 4 h prior to dosing for the fasting groups. Plasma samples were collected at 1, 2 and 168 h post-dose, and the data were normalized to the target protein levels at 1 h. To examine different PEs, all PE solutions were prepared by dissolving each PE in nuclease free water at 75 mM. Other than indicated, siRNA-1 was used as a tool compound to assess all the PD studies. Animal numbers and dosing regimen are provided in the legend of respective figure.

**Table 1.** Sequences and chemical modifications of the siRNAs and REVERSIRs

siRNAs or REVERSIRs	Target mRNA	Sequences (5′– 3′)*	Molecular weight
siRNA-1	*F12*	gsasaacuCfaAfUfAfaagugcuuua-G3	8752.05
		VPuAfaagCfacuuuauUfgAfguuucsusg	7650.14
siRNA-2	*F12*	gsasaacuCfaAfUfAfaagugcuususa	6996.09
		VPuAfaagCfacuuuauUfgAfguuucsusg	7650.14
siRNA-3	*TTR*	usgsggauUfuCfAfUfguaaccaaga-G3	8784.04
		VPuCfuugGfuuAfcaugAfaAfucccasusc	7596.14
siRNA-4	*TTR*	usgsggauUfuCfAfUfguaaccaaga-G3	8784.04
		usCfsuugGf(Tgn)uAfcaugAfaAfucccasusc	7494.11
siRNA-5	*F12*	gsasaacuCfaAfUfAfaagugcuuua-stabilized G3	8791.04
		VPuAfaagCfacuuuauUfgAfguuucsusg	7650.14
siRNA-6	*F12*	gsasaacucaauaaagugcuuua-G3	8800.13
		VPuaaagcacuuuauugaguuucsusg	7698.22
siRNA-7	*TTR*	asascaguGfuUfCfUfugcucuauaa-G3	8681.99
		usUfsauaGfagcaagaAfcAfcuguususu	7652.16
REVERSIR1	*TTR*	usgs(m5Cln)us(m5Cln)(Tln)as(Tlns)(Aln)dA-G3	5128.36
REVERSIR2	*TTR*	asasasascsasgsusgsususcsususgs(m5Clns) (Tlns)(m5Clns)(Tlns)as(Tlns)adA-G3	9749.76

*Sense strand sequences are the top rows; antisense strand sequences are the bottom rows. REVERSIRs are single strands. Structures and chemical modifications used for the synthesis of oligonucleotides in this table are shown in Figure 1. Lower case a, g, u and c represent 2′-OMe adenosine, guanosine, uridine, and cytidine, respectively. Af, Gf, Uf and Cf represent 2′-F adenosine, guanosine, uridine, and cytidine, respectively. (Tln), (Aln) and (m5Cln) represent LNA modifications of thymidine, adenosine, and 5-methylcytidine, respectively. Other chemical modifications are indicated as follows: s, phosphorothioate linkage; VP, 5′-(*E*)- vinylphosphonate; (Tgn), GNA modification of thymidine; Abbreviations: F12 = Factor 12; TTR = Transthyretin.

### PK/PD studies in Cynomolgus monkey

Cynomolgus monkey, *Macaca fascicularis* (2–4 kg, 3 animals/group), were dosed *via* either oral gavage or s.c. injection. The GalNAc–siRNAs (siRNA-3 or siRNA-4) in 150 mM C10 were given to NHPs *via* oral gavage using a syringe with an attached gavage tube at a dose volume of 5 ml/kg followed by tap water flush of 5 ml, while the same siRNAs in PBS were administered *via* s.c. at 1 ml/kg. For PD analysis, blood samples were collected at days 0 (pre-dose), 3, 7, 14, 21, 28, 35 and 42 post-dose. For plasma PK analysis, blood collection was conducted at pre-dose, and 15 min, 30 min, 1 h, 2 h, 4 h, 8 h, 12 h, 24 h and 48 h post-dose. Plasma samples were extracted using Clarity OTX^TM^ SPE 96-well plate cartridges, reverse transcribed to cDNA, and then quantified by stem-loop quantitative reverse transcription polymerase chain reaction (SL-qPCR) on a ViiA™ 7 Real-Time PCR system. For liver PK analysis, liver biopsy sample was collected from 1 animal/group/time point at day 0 (2 and 6 h post-dose), and days 1, 6, 7, 14, 21, 28 and 42 for a total of up to three non-terminal biopsies per animal. Samples were stored at –80°C until analysis. Liver lysates were extracted using cartridges as plasma samples, and antisense strand-based duplex concentration was quantified by liquid chromatography–high resolution mass spectrometry (LC–HRMS). Pharmacokinetic parameters for plasma and liver were calculated using a non-compartmental method in Phoenix WinNonlin 7.0. Oral bioavailability relative to s.c. dose was calculated with the equation (1) below:


(1) \begin{eqnarray*} && Bioavailability\ \left( {\% F} \right) = \nonumber\\ && \left( {AU{{C}_{p.o.}} \times Dos{{e}_{s.c.}}} \right)/(AU{{C}_{s.c.}}\ \times \ Dos{{e}_{p.o.}})\ \times \ 100\end{eqnarray*}

### *In vitro* metabolism assays

Stabilities of the GalNAc–siRNAs were assessed by incubating 50 μl of mouse, monkey, or human derived gastric or intestinal fluids (BioIVT) with 12.5 μl of GalNAc–siRNAs (0.5 mg/ml) in a 96-well plate for 6 h at 37°C with gentle shaking. Samples were then diluted with 450 μl lysis buffer (Phenomenex, Cat. No. AL0-8579) that was adjusted to pH 5.5 using ammonium hydroxide for solid phase extraction (SPE). SPE was then performed using Clarity OTX SPE plates (Phenomenex, Cat. No. 8E-S103-EGA). The plates were conditioned with 1 ml methanol using a Biotage ExtraHera and 1.9 ml equilibration buffer (50 mM ammonium acetate with 2 mM sodium azide, pH 5.5) prior to the sample loading. The column was washed with 1.5 ml wash buffer (50 mM ammonium acetate in 50% acetonitrile, pH 5.5) 5 times. Samples were eluted with 0.6 ml elution buffer (10 mM EDTA, 100 mM ammonium bicarbonate, 10 mM DTT in 40% acetonitrile and 10% THF, pH 8.8) and dried using nitrogen flow (TurboVap, 65 psi N_2_ at 40°C). After SPE, samples were reconstituted in 120 μl water and analyzed using liquid chromatography (Thermo Ultimate 3000) combined with mass spectrometry detection on a Thermo QExactive by heated electrospray ionization (HESI). Samples were injected (30 μl) and separated *via* Acquity UPLC using a BEH C8 column (Waters, Cat. No. 176002554) at 60°C. Mobile phase A was 0.1% diisopropylethylamine and 1% hexafluoroisopropanol in water, and mobile phase B was methanol. A gradient of 0–65% mobile phase B over 5.2 min was employed at 1 ml/min. The HESI source was operated in negative ion mode, with full scan, using spray voltage = 2800 V, sheath gas flow = 65 units, auxiliary gas flow = 20 units, sweep gas flow = 4 units, capillary temperature = 300°C, and auxiliary gas heated to 300°C. PromassHR (Novatia) software was used to deconvolute the signal.

### Quantification of liver siRNA levels in mice

Livers were collected at 6 h post-dose, snap-frozen in liquid nitrogen, pulverized into powder using a SPEX Genogrinder, and roughly ten milligrams were transferred to a tube for sample analysis. Tissues were lysed in Clarity OTX Lysis-Loading Buffer (Phenomenex) for a final concentration of 100 mg/ml. Samples were homogenized at 1000 rpm for 3 h at room temperature. Following homogenization, the samples were centrifuged at max speed for 15 min at 4°C. The cleared lysate was then diluted by 1:1000 in PBS with 0.25% Triton X-100. A 9-point standard curve was generated for each siRNA. Diluted samples and standards were heated at 95°C for 10 min. Immediately following heat denaturation, 5 μl of each sample was added directly to 10 μl of a stem–loop reverse transcription Master Mix (Applied Biosystems). Reverse transcription (16°C for 30 min, followed by 42°C for 30 min) and stop reaction (85°C for 5 min) were performed. The resulting complementary DNA (cDNA) was diluted in nuclease-free water to a final volume of 37.5 μl. SL-qPCR was carried out using an ABI ViiA 7 and QuantStudio software with an Applied Biosystems TaqMan Fast Advanced MasterMix in 20 μl reactions. The cycling parameters for the PCR were as follows: polymerase activation (95°C for 2 min), 40 cycles of PCR with a denaturing step (95°C for 1s), and annealing/extension (60°C for 20 s). The average cycle threshold (Ct) values of the 9-point standard curve were then used to form a linear regression to calculate the concentration of the siRNA present in each sample. The stem–loop primer, forward primer, reverse primer and probe sequences are listed in Supplement Table S1.

### *In vivo* reversal of siRNA activity using REVERSIR molecules

Female C57BL/6 mice aged 6–8 weeks received a single s.c. injection of siRNA-7 at 1 mg/kg (0.06 μmol/kg) at day 0. REVERSIR molecules (9-mer and 22-mer) in 150 mM C10 were orally dosed at day 7 at 0.1, 0.3, 1 and 3 mg/kg (equivalent to 0.02, 0.06, 0.19 and 0.58 μmol/kg, respectively) for the 9-mer molecule, and at 1, 3, 10 and 30 mg/kg (equivalent to 0.10, 0.31, 1.02 and 3.07 μmol/kg, respectively) for the 22-mer molecule. As a control group for each molecule, each REVERSIR molecule was dosed *via* s.c. injection at day 7 at 0.1 mg/kg (9-mer) and 0.3 mg/kg (22-mer), respectively. Plasma samples were collected at days 0 (pre-dose), 4, 8, 10, 12, 15 and 22, and analyzed for TTR protein level by ELISA assay.

## Results

### Robust *in vivo* activity in mice after oral administration of GalNAc–siRNA with C10 as PE

**Figure 2.**
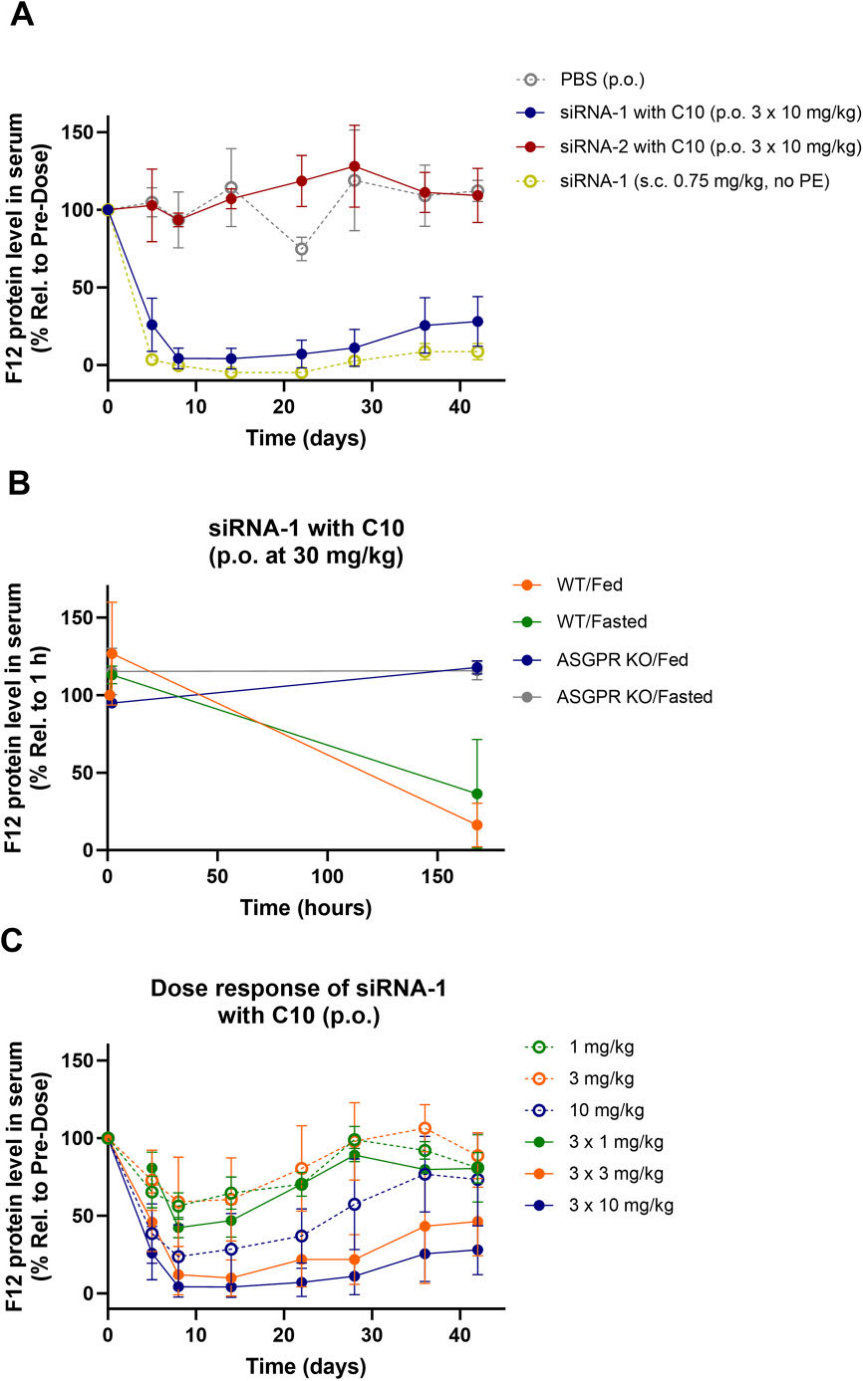
GalNAc–siRNA activity after oral administration in mice. (**A**) F12 targeting GalNAc–siRNA (siRNA-1), the siRNA of the same sequence/chemistry but without GalNAc (siRNA-2), and PBS were orally dosed at days 0, 2 and 5. The activity was compared with the group that received a s.c. injection of the siRNA-1. *n* = 3 for all groups except for siRNA-2 p.o. group (*n* = 2). (**B**) Comparison of GalNAc–siRNA activity in C57/BL6 WT and ASGPR KO mice received an oral gavage of siRNA-1 (*n* = 3). (**C**) Comparison of a single dose (at day 0) and three doses (at days 0, 2 and 5) of siRNA-1 after oral administration (*n*= 3). PBS (vehicle control) displayed in Panel A was run at the same time as the data displayed in Panel C. Data is represented as mean ± SD.

The siRNA conjugates and REVERSIRs are shown in Table 1. To explore oral delivery of GalNAc–siRNAs, we prepared GalNAc–siRNA (siRNA-1) targeting Factor 12 (F12) mRNA (21), co-formulated with C10. This oral dosing solution was stable without detectable aggregation or decomposition for more than 60 days and stored at 2–8°C until use. Following three doses of 10 mg/kg *via* oral administration, circulating F12 protein levels in serum were monitored at indicated time points for 42 days (Figure 2A). On day 5, circulating F12 protein levels were reduced by 74%, indicating orally administered GalNAc–siRNA was absorbed through GI and bioavailable in the liver. The siRNA activity at nadir reached ∼96% suppression of F12, which was comparable to a s.c. dose of 0.75 mg/kg and corresponded to a relative %F of 1.9%. As expected, no change in F12 protein levels was observed in the mice which received oral gavage of PBS or siRNA-2 with identical sequence/chemistry as siRNA-1 but without the GalNAc ligand, confirming that the GalNAc ligand was crucial for the orally administered siRNA to elicit efficient hepatic uptake, as demonstrated in earlier studies (16,22). This suggests that the GalNAc ligand exhibits sufficient metabolic stability during the oral absorption process for the uptake *via* ASGPR in the liver.

To further confirm ASGPR-mediated uptake, we compared the activity in wild-type (WT) and ASGPR knockout (KO) mice. As shown in Figure 2B, robust F12 protein reduction was observed only in WT and not in ASGPR KO mice, confirming ASGPR-mediated delivery. We also found that there were no significant differences in activity between fasted and fed states before dosing, indicating minimal impact of food intake on the oral absorption of GalNAc–siRNAs at the given dose level.

We assessed GalNAc–siRNA activity after single or multiple oral administrations. As shown in Figure 2C, oral administration of GalNAc–siRNAs led to F12 protein reduction in a dose-dependent manner. Consistent with our prior reports (23), orally administered GalNAc–siRNAs also demonstrated durable activity, suggesting intracellular trafficking following oral absorption is not significantly different from that observed in s.c. group. We found that mice receiving three doses of GalNAc–siRNA at 3 mg/kg showed better activity than the single dose of 10 mg/kg group. Similar trend of improvement was also seen in mice receiving three doses of GalNAc–siRNA at 1 mg/kg versus single dose of 3 mg/kg. The results suggest that a multi-dose regimen may elicit slightly improved activity than a single dose. Estimations of the relative bioavailability based on the observed F12 protein levels following oral administrations versus a s.c. injection at 0.75 mg/kg demonstrated a trend for decreased bioavailability with increasing dose levels, suggesting that additional optimization of the PE, the frequency of administration, and selection of dose level may further enable oral administration.

### Potency of the orally administered GalNAc–siRNAs depends on PEs

**Figure 3.**
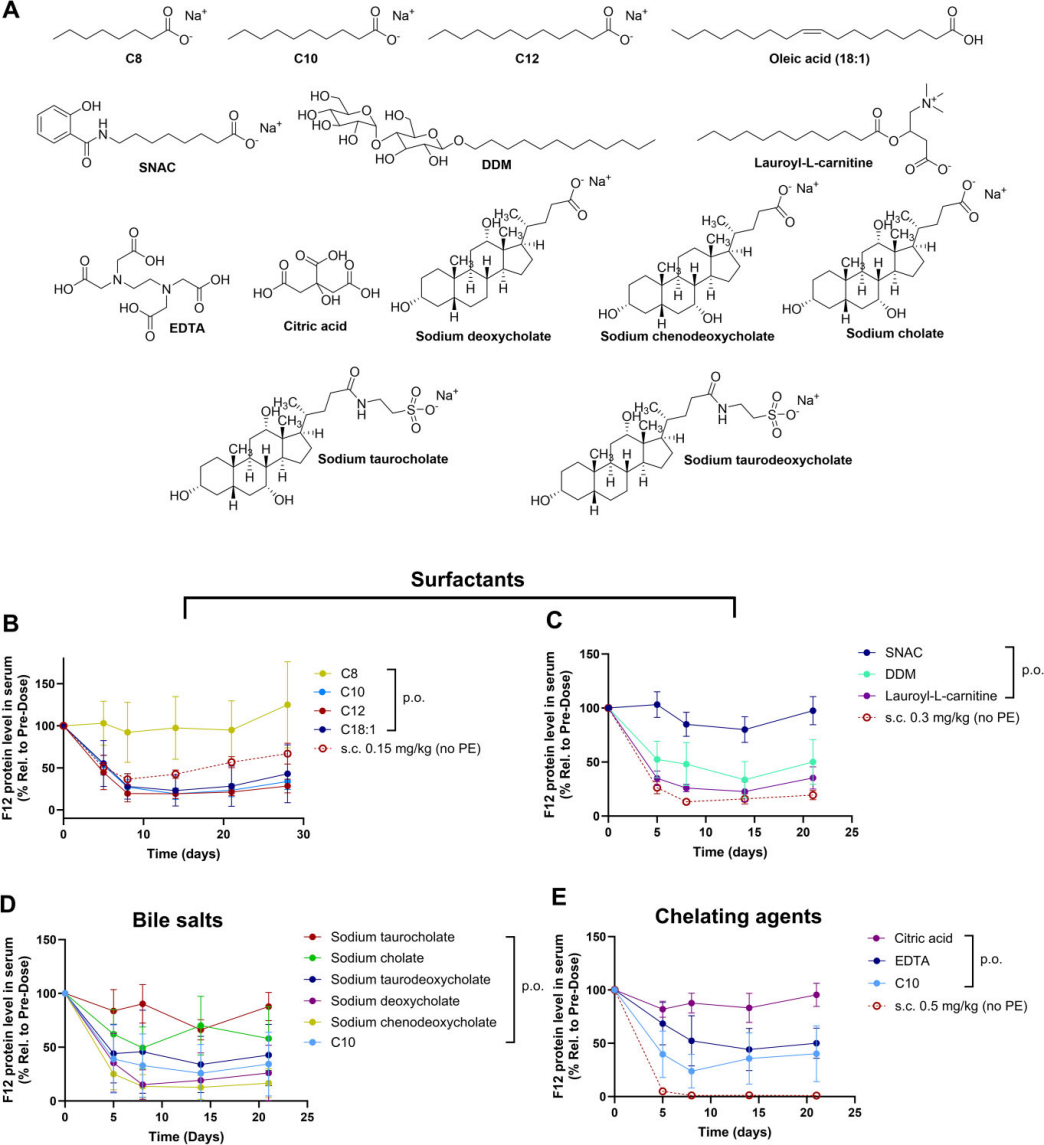
GalNAc–siRNA activity after oral administration with various PEs. (**A**) Structures of PEs tested. (**B–E**) Mice received oral gavage of GalNAc–siRNA (siRNA-1) formulated with different PEs (p.o. 3 × 3 mg/kg) or a single dose of siRNA-1 *via* s.c. injection. *n* = 4 for all groups except for sodium chenodeoxycholate group (*n* = 3). C10 benchmark formulation was employed as a control for evaluating each class of PE. Data is represented as mean ± SD.

PEs transiently increase the intestinal permeability of co-administered macromolecules *via* either paracellular or transcellular transports, or a combination of the mechanisms by altering mucosal barrier integrity. C10 is one of the most studied intestinal PEs for biologics such as peptides and nucleic acid-based modalities in the GI (24). After observing GalNAc–siRNA activity following oral administration with C10, we examined several additional classes of PEs, which were previously reported as potential absorption enhancers for oral delivery of proteins and peptides in preclinical or clinical settings (25,26). These included: (i) surfactants (sodium caprylate (C8), sodium laurate (C12), oleic acid (C18:1), salcaprozate sodium (SNAC), dodecyl-β-d-maltopyranoside (DDM), lauroyl-l-carnitine), (ii) chelating agents (ethylenediaminetetraacetic acid (EDTA), citric acid) and (iii) bile salts (sodium deoxycholate, sodium chenodeoxycholate, sodium cholate, sodium taurocholate, sodium taurodeoxycholate) as shown in Figure 3A.

We prepared oral formulations by reconstituting the GalNAc–siRNA (siRNA-1) in the PE solutions. Mice received oral gavage of GalNAc–siRNA formulated with different PEs at 3 mg/kg on days 0, 2 and 5. GalNAc–siRNA with the C10 benchmark formulation consistently led to robust and durable target knockdown across animal studies. As shown in Figures 3B–E, most of the tested formulations supported some level of target knockdown but not with PEs such as C8, SNAC, citric acid, and sodium taurocholate. The results indicate that the transport of GalNAc–siRNAs across the intestinal mucosal barrier depends on the type of PE used in the formulation. Though C12 and C18:1 showed comparable or slightly better activity than C10, they required additional heating for solubility and thus were excluded from further assessment.

**Figure 4.**
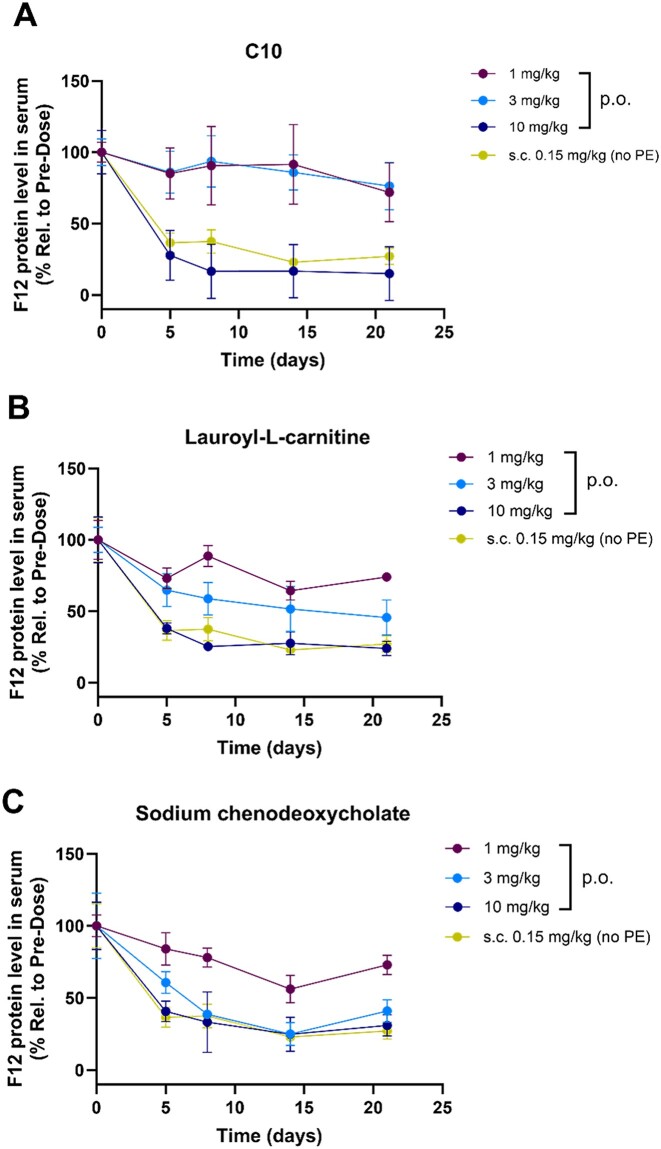
Dose responses of orally administered GalNAc–siRNA (siRNA-1) formulated with (**A**) C10, (**B**) lauroyl-l-carnitine and (**C**) sodium chenodeoxycholate in mice. *n* = 4 for all groups except for 3 mg/kg lauroyl-l-carnitine and 1 or 10 mg/kg sodium chenodeoxycholate groups (*n* = 3). The activity was compared with a s.c. dose of siRNA-1 (*n* = 4).

Lauroyl-L-carnitine and sodium chenodeoxycholate were selected for further evaluation based on the initial data. As shown in Figure 4A-C, a single dose of GalNAc–siRNA formulated with lauroyl-l-carnitine or sodium chenodeoxycholate at 1 mg/kg and 3 mg/kg, respectively, showed improved knockdown than that formulated with C10 at the same dose levels. However, durable target knockdown with a maximum suppression of 83% was achieved with C10 compared to 75–77% for other PEs. Given that utilizing C10 consistently demonstrated robust knockdown and durability in our studies, and it has been tested in humans previously as one of the most advanced PEs for poorly permeable drugs (27,28), we selected C10 as a proof-of-concept PE for further PK/PD studies in higher species.

### Orally administered GalNAc–siRNAs are potent and durable in NHPs, but show limited bioavailability

**Figure 5.**
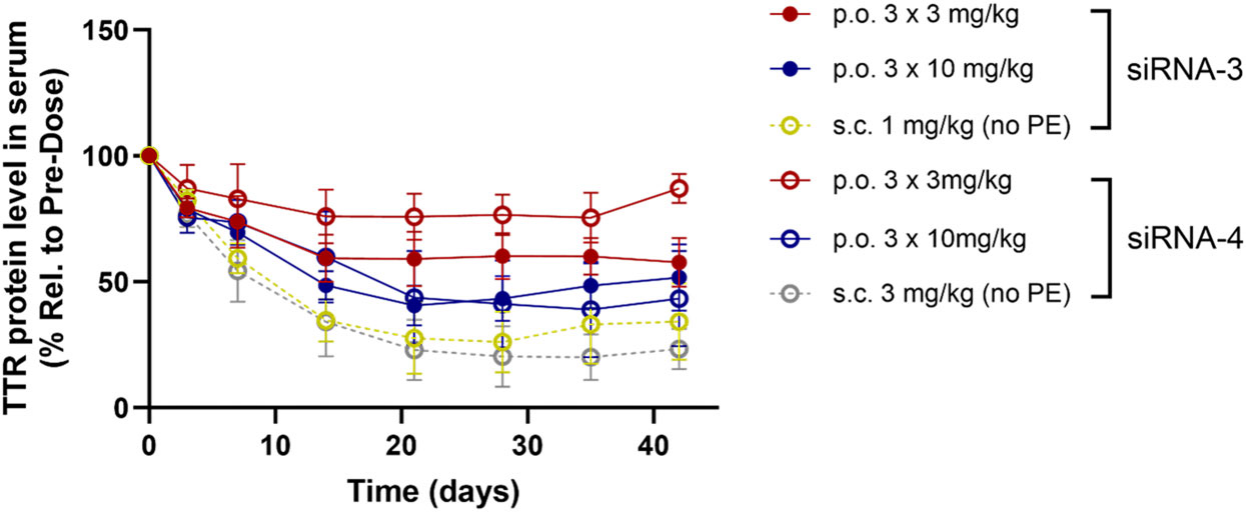
Translation of orally administered GalNAc–siRNA activity in NHP. Cynomolgus monkeys received oral gavage of formulation containing TTR GalNAc–siRNAs (siRNA-3 or siRNA-4) and C10 (150 mM) at days 0, 2 and 5. The same GalNAc–siRNAs without PE were dosed *via* s.c. injection as a control for each GalNAc–siRNA group. Plasma samples were collected at indicated days for circulating TTR protein measurement relative to pre-dose. Data is represented as mean ± SD, for *n* = 3.

Next, we assessed whether the activity seen after oral administration of GalNAc–siRNAs formulated with C10 in mice would translate to NHPs. We used GalNAc–siRNAs (siRNA-3 and siRNA-4) targeting the same site in transthyretin (TTR) mRNA with different chemical modifications for the NHP study (29,30). Cynomolgus monkeys were dosed with oral formulations of GalNAc–siRNAs through oral gavage tube and plasma samples were collected to monitor circulating TTR protein levels at different time intervals. As shown in Figure 5, TTR protein levels were reduced by up to 42% and 25% at a total dose of 9 mg/kg and 60% and 61% at a total dose of 30 mg/kg after oral administrations of siRNA-3 and siRNA-4, respectively. However, a single s.c. injection (1–3 mg/kg) of these GalNAc–siRNAs resulted in more robust knockdown, 74% and 80%, respectively, than when orally administered at 10 to 30-fold higher doses. Although knockdown levels were different, comparable PD profiles of TTR protein reduction for either route of administration suggest that intracellular depot for the extended duration of GalNAc–siRNA activity observed in s.c. groups (23) is maintained even after oral administration. During the study, oral administrations of the GalNAc–siRNAs with C10 were well-tolerated in NHPs without any clinical observations.

**Figure 6.**
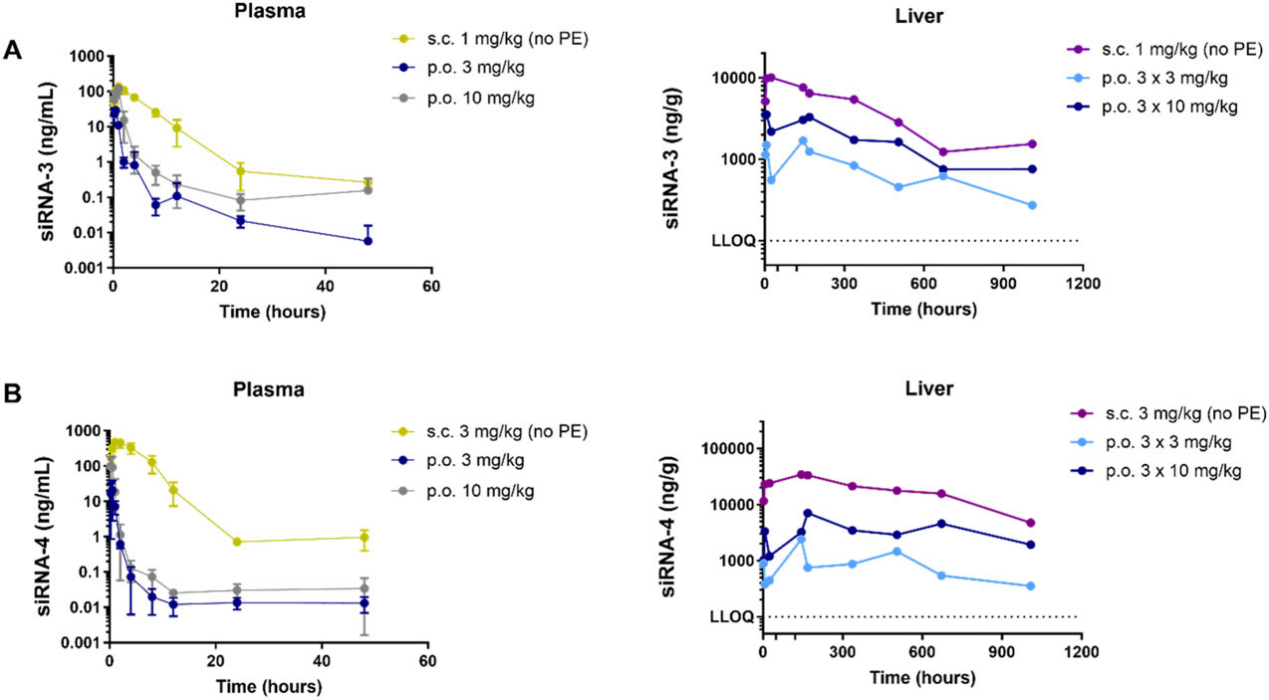
Plasma and liver concentration-time profiles of the GalNAc–siRNAs (siRNA-3) (**A**) and (siRNA-4) (**B**) in NHPs. Blood was collected from the cynomolgus monkeys after oral gavage at indicated time points for the quantification of plasma siRNA levels. Liver biopsy was conducted from 1 animal/group/time point for the quantification of liver siRNA levels. A single dose of s.c. administration of the same GalNAc–siRNA was used for comparison. Plasma PK values are represented as mean ± SD, for *n* = 3. Liver PK values are represented as individual data.

Plasma PK profiles of GalNAc–siRNAs (siRNA-3 and siRNA-4) in NHPs were evaluated using SL-qPCR (Figure 6 and Table 2). Following a single oral administration of siRNA-3 or siRNA-4, the absorption into systemic circulation was rapid with T_max_ of 0.25–1 h for siRNA-3 and 0.25–0.5 h for siRNA-4, which was faster than that shown in the corresponding s.c. groups. The plasma exposure of orally administered siRNA-3 indicated an approximately dose-proportional increase in *C*_max_ from 31.2 ng/ml to 120.0 ng/ml, and a slightly greater than dose-proportional increase in AUC_last_ from 30.1 h*ng/ml to 174.0 h*ng/ml at 3 and 10 mg/kg, respectively. The relative bioavailability in plasma following oral administration was 1.4–2.5%. Similar to siRNA-3, siRNA-4 showed an approximately dose-proportional increase in plasma *C*_max_ and AUC_last_ between 3 and 10 mg/kg following oral administration. The plasma bioavailability for the siRNA-4 was however lower, 0.7–0.8%.

**Table 2.** Summary of plasma and liver PK parameters in NHPs following s.c. or p.o. doses of GalNAc–siRNAs (siRNA-3 and siRNA-4)

**Plasma PK**	**siRNA**	**Dosing route**	**Dose level (mg/kg)**	***T*_max_ (h)**	***C*_max_ (ng/ml)**		AUC_last_ (h*ng/ml)	**%F AUC_last_**
	siRNA-3	p.o.	3	0.25–0.5	31.2 ± 4.2		30.1 ± 3.9	1.4
		p.o.	10	1	120.0 ± 36.6		174.0 ± 58.3	2.5
		s.c.	1	1	138.0 ± 10.3		709.0 ± 151.0	N.A.
	siRNA-4	p.o.	3	0.25–0.5	21.5 ± 16.6		19.0 ± 13.1	0.7
		p.o.	10	0.25–0.5	112.0 ± 89.4		76.0 ± 70.7	0.8
		s.c.	3	1–2	508.0 ± 114.0		2890.0 ± 348.0	N.A.
**Liver PK**	**siRNA**	**Dosing route**	**Dose level (mg/kg)**	***t*_1/2_ (h)**	***T*_max_ (h)**	***C*_max_ (μg/g)**	AUC_last_ (h*μg/g)	**%F AUC_last_**
	siRNA-3	p.o.	3	416	144	1.7	719	2.0
		p.o.	10	382	6	3.6	1610	1.4
		s.c.	1	328	24	10.1	3940	N.A.
	siRNA-4	p.o.	3	267	144	2.4	869	1.5
		p.o.	10	913	168	7.0	3570	1.9
		s.c.	3	319	144	34.2	18800	N.A.

Abbreviations: N.A. = not applicable; *T*_max_= time to reach maximum concentration; *C*_max_= maximum observed concentration; AUC_last_= area under the concentration-time curve from the time of dosing to the last measurable concentration; %F AUC_last_= bioavailability based on AUC_last_; *t*_1/2_= elimination half-life. Plasma *C*_max_ and AUC_last_ values are represented as mean ± SD, for *n* = 3. Liver PK parameters are represented as individual data.

Liver PK parameters for the p.o. groups were analyzed after administrations of GalNAc–siRNAs at indicated dose levels on days 0, 2 and 5.

To investigate the liver PK, liver biopsies were performed from 1 animal/group/time point at the indicated time points (Figure 6A and B) and siRNA concentrations were then measured *via* LC-HRMS. Since GalNAc–siRNA is hepatocyte targeted, distribution to the liver during first pass after oral administration serves as a favorable condition for improving exposure in the liver. As expected, the siRNA-3 and siRNA-4 following oral administration primarily accumulated in liver like those administered *via* s.c. injection (Figure 6 and Table 2). The *t*_1/2_ in liver were 382–416 and 267–913 h for the orally administered siRNA-3 and siRNA-4, respectively. The liver exposures of siRNA-3 and siRNA-4 exhibited dose-proportional increase in AUC_last_ between 3 and 10 mg/kg following oral administration. The relative oral bioavailabilities in liver for the siRNA-3 and siRNA-4 were 1.4–2.0% and 1.5–1.9%, respectively. There was a trend for higher calculated relative bioavailability when using siRNA exposure in liver rather than plasma; additionally, when using the PD observations as a surrogate for exposure, the relative bioavailabilities ranged from 3 to 10%. This may suggest improved GalNAc–siRNA uptake or differences in zonal distribution in liver following oral administration (30).

### Pre-systemic metabolism of GalNAc–siRNAs in GI is not significant

**Figure 7.**
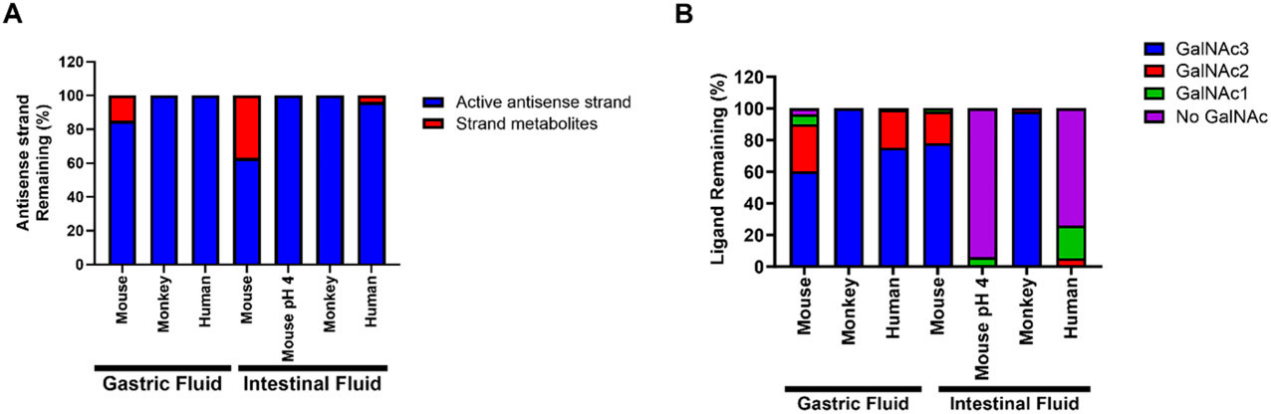
*In vitro* metabolic stability of GalNAc–siRNA (siRNA-1) in GI matrices of mouse, monkey, and human. Antisense strand (**A**) and GalNAc ligand in sense strand (**B**) metabolisms were analyzed in different GI matrices using LC-MS.

To investigate the key limiting factors for oral absorption of GalNAc–siRNAs, we first focused on pre-systemic degradation in the GI as it may significantly lower the bioavailability (31). The GalNAc–siRNA (siRNA-1) was incubated with gastric or intestinal fluids of different species (mouse, monkey, and human) for 6 h at 37°C and analyzed *via* LC-MS. Considering relatively short transit time of the GalNAc–siRNAs in the GI, 6 h represent exaggerated conditions for metabolic stability. When analyzing antisense strands, both full length and N-1 metabolite at the 3′-end (a nucleotide loss at the 3′-end of the antisense strand) were considered as active strands as this has been demonstrated previously across our siRNA conjugate platform for multiple targets in different species (32). The remaining active antisense strand in the mouse gastric and intestinal fluids after the 6 h incubation was about 85% and 63%, respectively, while negligible strand metabolites were found in higher species GI matrices (Figure 7A).

Next, we evaluated the stability of GalNAc ligand attached to the sense strand in GI matrices. As metabolites, di, mono, and no GalNAc metabolites are generated *via* glycosidic bond cleavage by glycosidases. In mouse and human gastric fluids, we observed approximately 20–40% cleavage of GalNAc ligand whereas it was stable in monkey gastric matrix (Figure 7B). Surprisingly, substantial cleavage of GalNAc ligand was observed in mouse intestinal fluids where the pH was adjusted to 4 to mimic the pH gradient of the GI tract and in human intestinal fluids. To investigate potential variability of the metabolism from human GI fluids, we tested both antisense strand and GalNAc ligand metabolisms of GalNAc–siRNA (siRNA-1) in human GI fluids from additional donors (Supplement Figure S1). Slight variability in nuclease activity of the human gastric fluids was detected among different donor fluids whereas noticeable variability was found in the human intestinal fluids. On the other hand, a large variability in glycosidase activity was observed in both human gastric and intestinal fluids. This variability is presumably due to the inability to control multiple parameters including health, diet, and fasted states of human donors prior to the sample collection, while other species such as rodent and monkey used for the metabolism analysis were healthy and on controlled diets.

**Figure 8.**
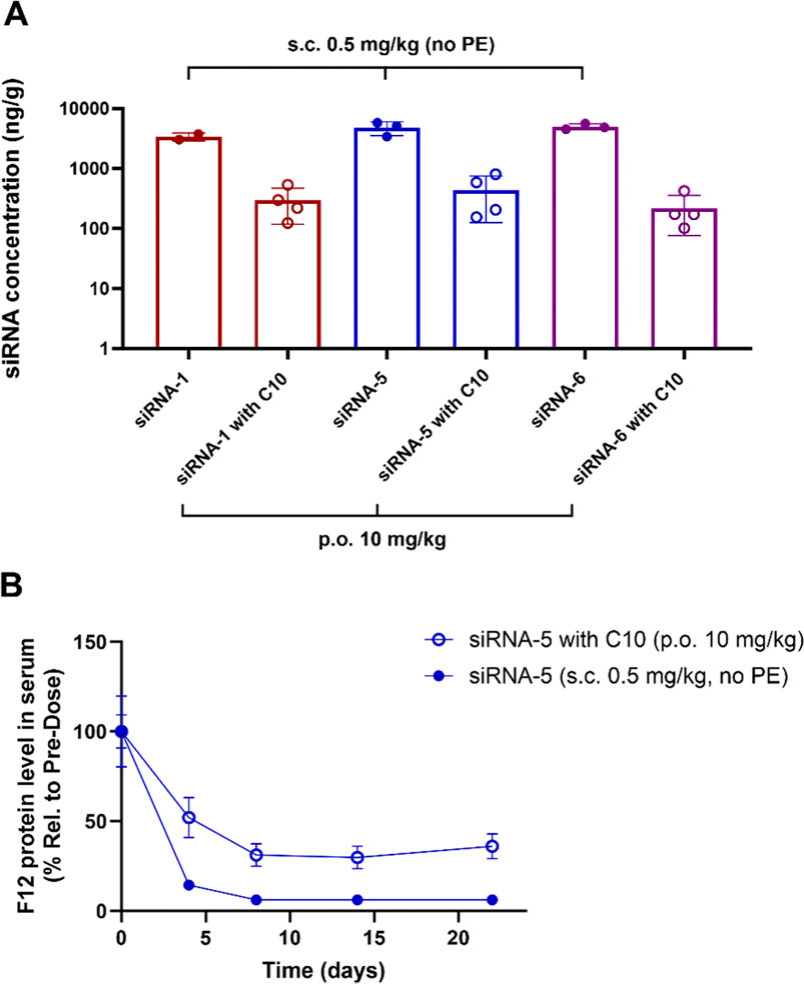
*In vivo* investigation of the stabilized versions of GalNAc–siRNAs in mice. (**A**) Comparison of the liver exposures for parent (siRNA-1), stabilized G3 modification (siRNA-5), and full 2′*O*Me modification (siRNA-6) after s.c. or p.o. administrations in mice. siRNA concentration was assessed by SL-qPCR (*n* = 2 for siRNA-1 s.c. group; *n* = 3 for siRNA-5 and siRNA-6 s.c. groups; *n* = 4 for all p.o. groups). (**B**) Serum F12 protein level after p.o. or s.c. administration of siRNA-5 in mice (*n* = 3 for s.c. group; *n* = 4 for p.o. group). Data is represented as mean ± SD.

To further explore the stability of the GalNAc–siRNAs, we investigated whether a metabolically stable glycosidic linkage containing GalNAc ligand (anomeric oxygen at the glycosidic bond was replaced by nitrogen: stabilized G3 shown in Figure 1) (33) or chemical modifications could improve liver exposure after oral administration. Three different versions of GalNAc–siRNAs were tested in mice: a parent compound (siRNA-1), a stable GalNAc version (G3 of the parent was substituted with stabilized G3) (siRNA-5), and a fully 2′*O*Me-modified version which represents a highly stable backbone against nuclease activity (siRNA-6) but lacks RNAi activity (Figure 1 and Table 1) (34). The GalNAc–siRNAs were dosed in mice *via* an oral gavage at 10 mg/kg with C10, or s.c. injection at 0.5 mg/kg. At 6 h post-dose, liver exposures were significantly higher in s.c. groups than in p.o. groups similar to the PK results in NHPs (Figure 8A). Administration of siRNA-5 or siRNA-6 *via* s.c. injection resulted in comparable or slightly increased liver exposure (approximately 1.4-fold increase in mean value) compared to siRNA-1, whereas no noticeable amelioration in liver exposure was found after oral administrations of siRNA-5 and siRNA-6. In accordance with the liver exposure, a single oral administration of the siRNA-5 containing the metabolically stabilized G3 ligand achieved a maximum target F12 protein reduction of approximately 70%, comparable to the level of activity observed from siRNA-1 with the parent ligand (Figure 8B and Supplement Figure S2). Given these *in vitro* and *in vivo* results, we conclude that neither GalNAc ligand cleavage nor antisense strand degradation in GI have any significant impact on bioavailability after oral administration.

### Shorter oligonucleotides show better intestinal permeability after oral administration with C10

To understand the impact of GalNAc–siRNA size on intestinal membrane permeability, we designed a study comparing activity of the oligonucleotides with different lengths and sizes after oral administration with C10. Previously, we have reported the reversal of GalNAc–siRNA-mediated RNAi activity with a single s.c. dose of GalNAc ligand conjugated oligonucleotides complementary to the antisense strand of GalNAc–siRNA, known as the REVERSIR technology (18). We decided to evaluate TTR REVERSIR molecules of two sizes – a 9-mer (5128 Da) and a 22-mer (9749 Da).

**Figure 9.**
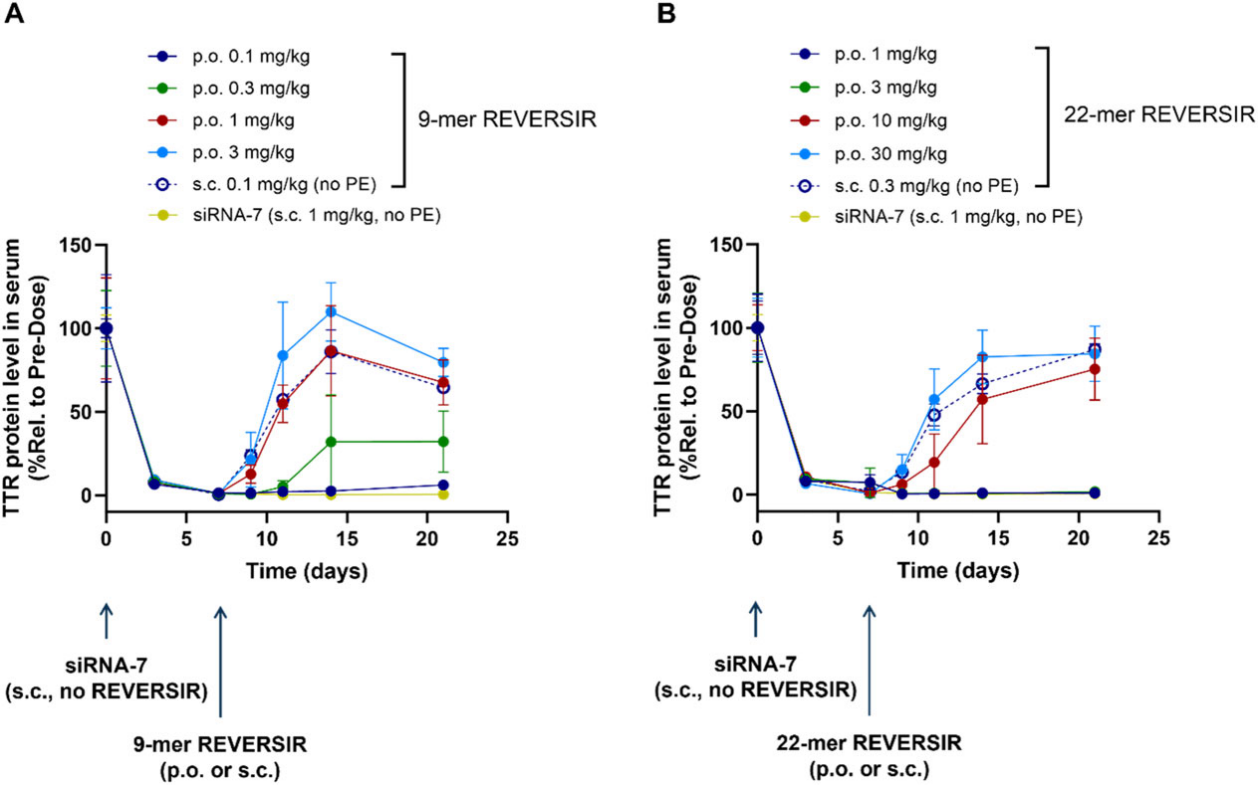
Comparison of reversal of GalNAc–siRNA activity for the 9-mer and 22-mer REVERSIR molecules after oral administration. Mice received a single s.c. injection of TTR targeting siRNA-7 at day 0 to achieve robust knockdown (*n* = 4). On day 7, mice received an oral gavage of (**A**) 9-mer TTR REVERSIR (*n* = 4 for all groups except for 0.1 mg/kg 9-mer REVERSIR p.o. group – *n* = 3) or (**B**) 22-mer TTR REVERSIR (*n* = 4 for all groups except for 3 mg/kg 22-mer REVERSIR p.o. group – *n* = 2). TTR protein levels were monitored to determine reversal activity of the different REVERSIR molecules administered *via* p.o. compared to their s.c. groups (*n* = 3). Individual graph for the overlapped data (siRNA-7 and 22-mer REVERSIR p.o. 3 mg/kg) in (B) is indicated in Supplement Figure S3. Data represented as mean ± SD.

We hypothesized that longer-length REVERSIR molecules would require higher dose than the shorter-length REVERSIR molecules for p.o. groups to achieve equivalent activity to the corresponding s.c. groups given that larger molecules have more challenges in intestinal permeability. To achieve target mRNA knockdown, we dosed GalNAc–siRNA (siRNA-7) targeting TTR to mice *via* a single s.c. injection. After that, either a single s.c. injection or an oral gavage of TTR REVERSIR molecules were dosed to mice on day 7 to initiate reversal of TTR silencing activity (Figure 9 and Supplement Figure S3). As in the s.c. groups, oral administration of 9-mer and 22-mer REVERSIR molecules reversed TTR knockdown. Orally dosed 9-mer REVERSIR at 1 mg/kg showed comparable activity to the same REVERSIR dosed *via* s.c. at 0.1 mg/kg, indicating a 10-fold lower relative bioavailability for the p.o. route. In contrast, orally dosed 22-mer REVERSIR at 10 mg/kg was inferior and 30 mg/kg exhibited slightly better or comparable to the same REVERSIR dosed *via* s.c. at 0.3 mg/kg, indicating an almost 30–100-fold lower relative bioavailability for the oral route. This data supports the idea that intestinal membrane permeability can be a challenging barrier for larger size macromolecules even in the presence of PEs. Further studies determining liver exposure of each REVIERSIR molecule for both administration routes may help to rule out the impact of dose level on bioavailability.

## Discussion

Currently available RNAi therapeutics are given by parental route with infrequent dosing intervals (few weeks to months). Here, we investigated whether the significant improvement in potency and durability of our current siRNA designs may allow delivery *via* oral route at practical dosage levels. Our findings show that oral administration of GalNAc–siRNAs is feasible in rodents and NHPs and elicits robust activity albeit at much higher doses compared to s.c. administration route.

Crossing the intestinal epithelial barrier in which tight junctions connect enterocytes is challenging for polyanionic macromolecules such as siRNAs, which also feature a rigid double helical structure. To enable intestinal absorption of GalNAc–siRNAs, we evaluated a few classes of intestinal PEs such as surfactants, chelating agents and bile salts which were previously reported as enhancers for oral delivery of macromolecular drugs. Most of the tested PEs including C10, lauroyl-L-carnitine, and sodium chenodeoxycholate facilitated oral absorption of the GalNAc–siRNAs in mice and resulted in robust RNAi activity. Moreover, a similar duration of RNAi activity was observed in animals administered *via* s.c. and p.o., suggesting that the orally administered GalNAc–siRNAs were intact when reaching the liver. Given the reported properties of these PEs (24–26,35), we attribute intestinal absorption of the GalNAc–siRNAs to the PE-mediated paracellular transport by transient modulation of the tight junction permeability, or in combination with the transcellular transport *via* temporary increase of intestinal membrane fluidity. Further evaluation of each PE utilized for the oral delivery of GalNAc–siRNAs will be required to determine their mechanism of action, optimal concentration, and tolerability. Among the most potent PEs, we selected C10 for additional PK/PD and metabolism studies as it is approved by FDA as a food additive with no limit for daily intake (27,36), and one of the most advanced PEs evaluated in the clinical trials for the oral administration of macromolecules (28,37).

Consistent with the mice data, potent and sustained silencing was observed in NHPs following oral administration of the GalNAc–siRNAs with C10. Plasma *T*_max_ for the p.o. groups were determined at 0.25–1 h which were equal to or faster than those in the s.c. groups, reflecting rapid intestinal absorption of the GlaNAc-siRNAs through the oral formulation containing C10. Given the rapid absorption after an oral administration of semaglutide tablet containing SNAC as a PE (38,39), immediate absorption *via* these PEs was not surprising. Significantly longer *t*_1/2_ values in the liver support that majority of the orally absorbed GalNAc–siRNAs are distributed to the liver as observed with the s.c. injection. However, the bioavailability in liver after oral delivery remained low, 1.4–2.0%, for both GalNAc–siRNAs tested.

Stability in the GI tract is one of the key properties to achieve high bioavailability after oral delivery of macromolecules. Oral peptide drugs approved or under clinical evaluations have been formulated in enteric-coated tablets with PEs to prevent their degradation in GI fluids and improve local concentration at the site of absorption (38,39). In addition, chemically modified ASOs tested across different species including human were formulated either in a single-unit or multiple enteric-coated tablets as oral dosage forms (11,13). The use of enteric-coated tablets can minimize degradation and enhance absorption, though the enhancement can still be impeded by a few factors such as insufficient PE concentration *via* spreading in the GI environment and an unoptimized formulation condition. We investigated siRNA degradation, GalNAc ligand cleavage, and liver exposure in various *in vitro/in vivo* conditions. *In vitro* evaluation in the GI fluids across different species showed that the intact or N-1 (active) antisense strands were the major species in mouse, monkey, and human GI matrices even after 6 h incubation, while degradation of the GalNAc ligand was dependent on pH and species. The rather long incubation with the GI matrices used in our experimental conditions likely exaggerate the metabolism, which will occur during the much shorter exposure of the formulated conjugates to GI fluids using enteric-coated tablets with rapid release kinetics in the intestine. The fact that minimal to no metabolites were observed in all monkey GI matrixes implies that metabolic stability of the GalNAc–siRNAs after oral administration is not the rate-limiting factor. Further, GalNAc–siRNA containing the ligand G3 with stable GalNAc-linkages (siRNA-5) maintained intact ligand under the same *in vitro* experimental conditions including human intestinal matrix but did not show increased liver exposure nor improved silencing activity compared to the parent siRNA-1. Similarly, siRNA-6, which was fully modified with 2′*O*Me and exhibited a more favorable metabolism profile in GI fluids than the parent (data not shown), did not show improved liver exposure. Taken together, it is unlikely that GalNAc ligand or siRNA metabolism plays a significant role for the observed relatively low liver exposure following oral administration and point towards other factors, such as intestinal permeability as more critical parameters for oral bioavailability of GalNAc–siRNAs.

Compared to the approved oral peptide drugs, GalNAc–siRNAs have an approximately 4 to 16-fold higher molecular weight. Previously, oral administration of chemically modified ASO (roughly half the size of siRNA), co-formulated with C10 using multiple tablets, showed plasma bioavailability of up to 10% in humans (11). And a recent report on daily oral administration of the GalNAc-linked 16-mer ASO in a single tablet indicates feasibility of oral administration; however, the bioavailability remains in single digits (40). Given the low bioavailability and need for high amount of PE, the size and number of tablets for oral dosage could still be a significant burden. To understand the role of oligonucleotide size on GI permeability, we utilized REVERSIR molecules with different sizes (9-mer versus 22-mer). Interestingly, the larger-sized REVERSIR required a significantly greater amount for the p.o. groups than the smaller one to reach the reversal activity observed in the corresponding s.c. groups. Given the duplex nature of siRNAs with a molecular weight of ∼16 kDa, approximately 1.7-fold larger than the 22-mer REVERSIR, the size and rigidity of the siRNA molecules can be considered as the key limiting factor for oral delivery.

This is the first report of systematic evaluation of the oral delivery of GalNAc–siRNA in pre-clinical species. Our results suggest that intestinal permeability is the biggest challenge for oral absorption of GalNAc–siRNAs, and this may be due to large size and rigid double-stranded structure of siRNA. Therefore, additional work will be needed to achieve effective permeability along with high bioavailability to enable potential clinical development. Continued effort in this space has potential to yield the ‘conventional route’ of dosing for such ‘modern medicines’ and have even broader impact.

## Supplementary Material

gkae350_Supplemental_Files

## Data Availability

The authors declare that all data supporting the findings of this study are available within the article and the Supplementary Information files.
