# Supplementary material for: Evaluating the oral delivery of GalNAc-conjugated siRNAs in rodents and non-human primates
Source: Nucleic Acids Res. 2024 May 14;52(10):5423–37. doi: 10.1093/nar/gkae350 (PMC11162796; doi:10.1093/nar/gkae350)
Supplement: gkae350_Supplemental_Files [file gkae350_supplemental_files.zip › Supplementary Material_Final_revised.pdf]

Supplementary Material of:

Evaluating the oral delivery of GalNAc-conjugated siRNAs in rodents and non-human primates

Mikyung Yu[1], June Qin[1], Xiumin Liu[1], Diane Ramsden[1], Brian Williams[1], Ivan Zlatev[1], Dale Guenther[1], Shigeo Matsuda[1], Roxanne Tymon[1], Justin Darcy[1], Catrina Wong[1], Jamie Tsung[1], Peter Zawaneh[1], Saeho Chong[1], Christopher S. Theile[1], Nathan Taneja[1], Arlin Rogers[1], Ju Liu[1], Elena Castellanos-Rizaldos[1], Sarah Bond[1], Kawai So[1], Jason Denoncourt[1], Adam Castoreno[1], Muthiah Manoharan[1], Jing-Tao Wu[1], Kevin Fitzgerald[1], Martin A. Maier[1], Vasant Jadhav[1], Jayaprakash K. Nair[1*]

[1]Alnylam Pharmaceuticals, Inc., Cambridge, MA 02142, USA

Supplement Table 1. Reverse transcription-quantitative polymerase chain reaction primer and probe sequences

**Primer**	**Sequence (5'-3')**
Stem-loop	GTCGTATCCAGTGCAGGGTCCGAGGTATTCGCACTGGATACGACCAGAAACTCA
Forward	GCCCGTAAAGCACTTTATTG
Reverse	GTGCAGGGTCCGAGGT
Probe	TGGATACGACCAGAAAC

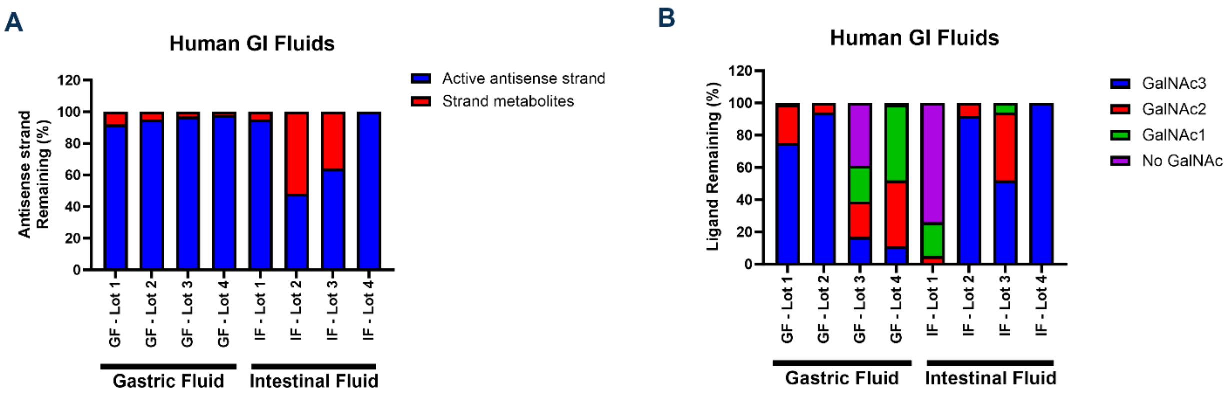

Supplement Figure 1. *In vitro* metabolic stability of the GalNAc-siRNA (siRNA-1) in human GI matrices from different donors. Antisense strand (**A**) and GalNAc ligand in sense strand (**B**) metabolisms were analyzed in different GI matrices using LC-MS.

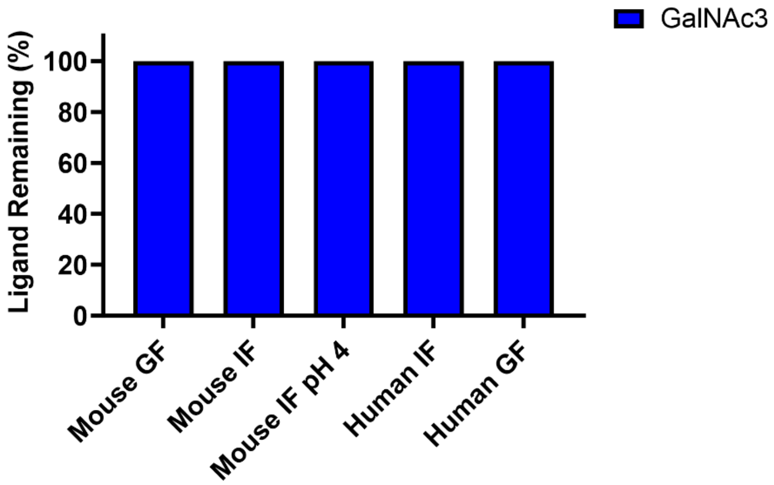

Supplement Figure 2. *In vitro* metabolism of the GalNAc-siRNA containing the stabilized G3 (siRNA-5) in mouse and human GI matrices, analyzed by LC-MS.

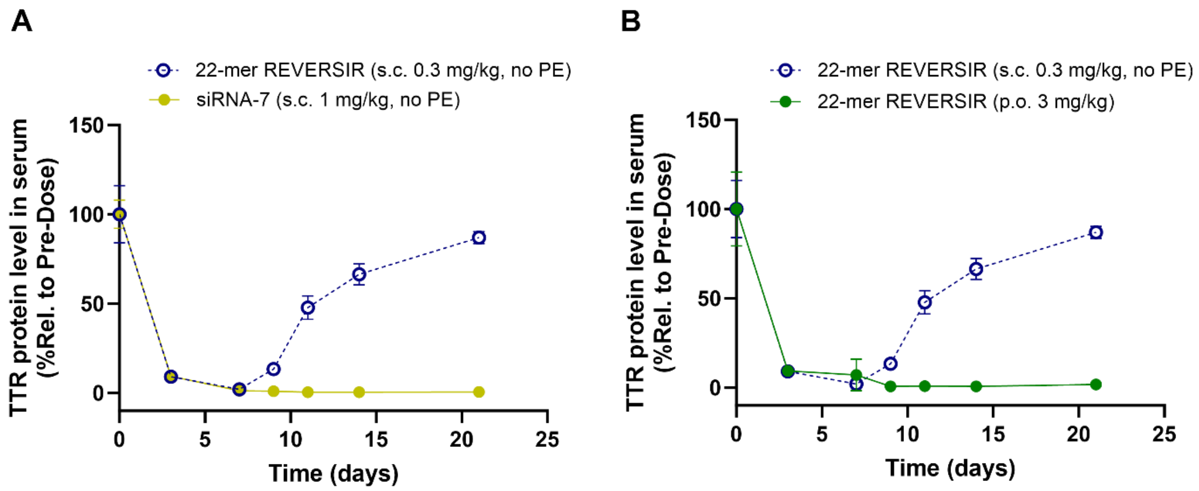

Supplement Figure 3. Individual graph for the siRNA-7 (s.c. 1 mg/kg) (**A**) and 22-mer REVERSIR (p.o. 3 mg/kg) (**B**) from Figure 9B. 22-mer REVERSIR (s.c. 0.3 mg/kg) is shown for comparison.
